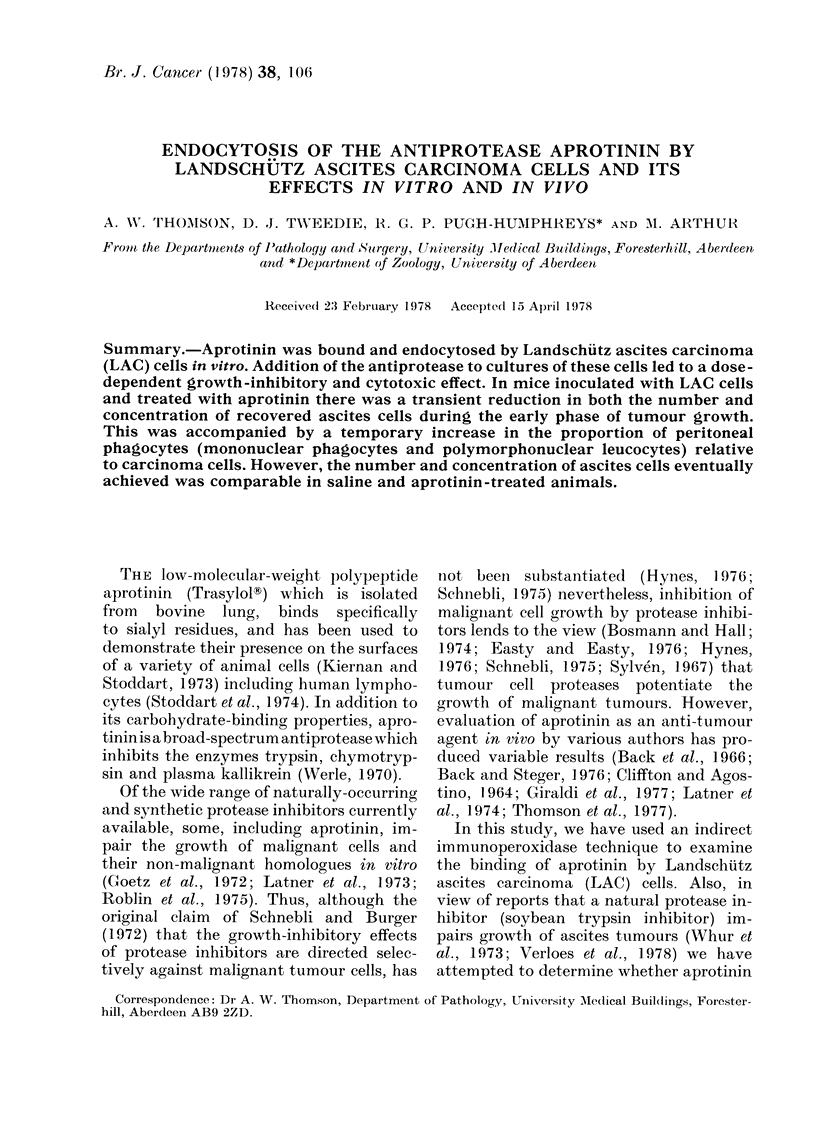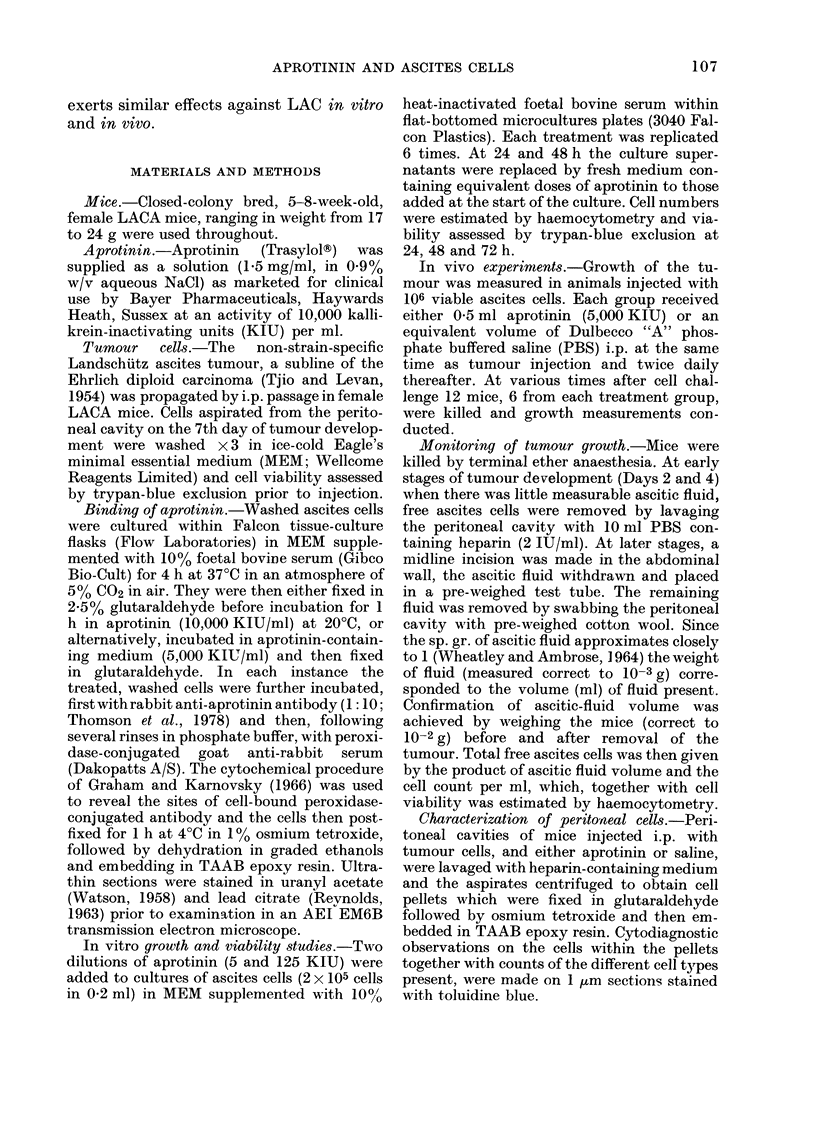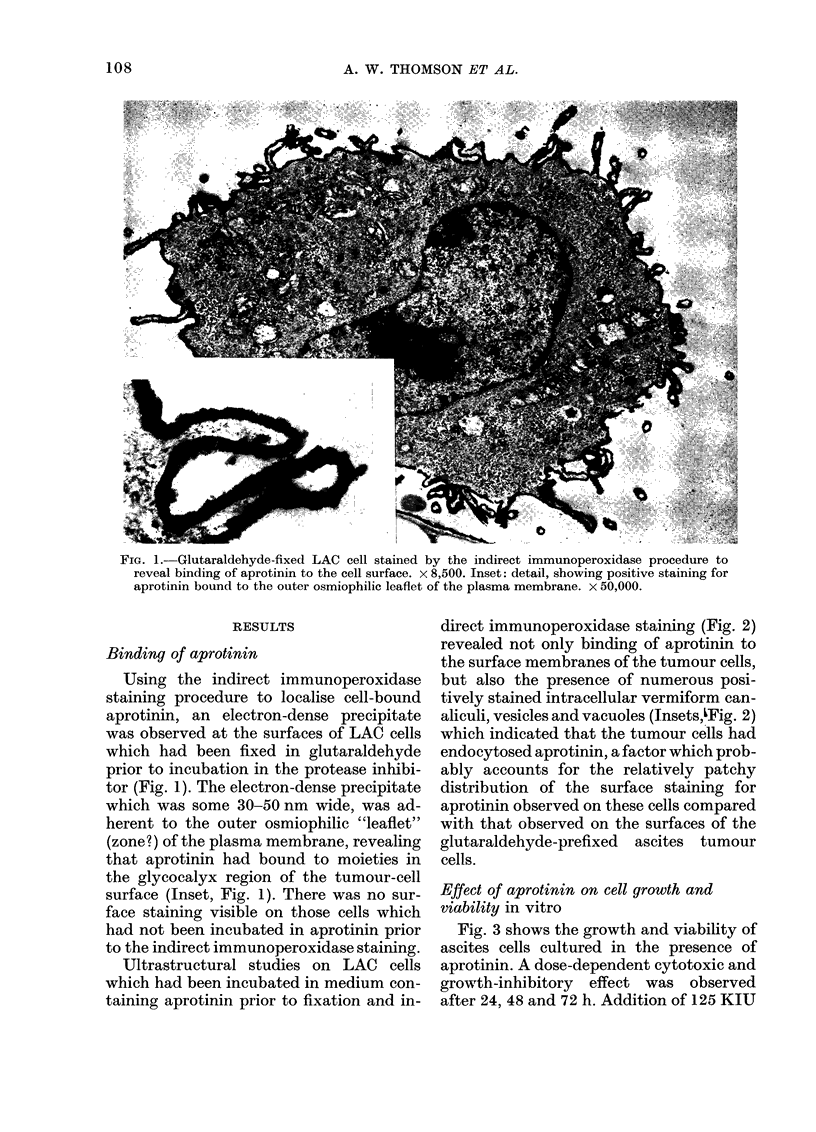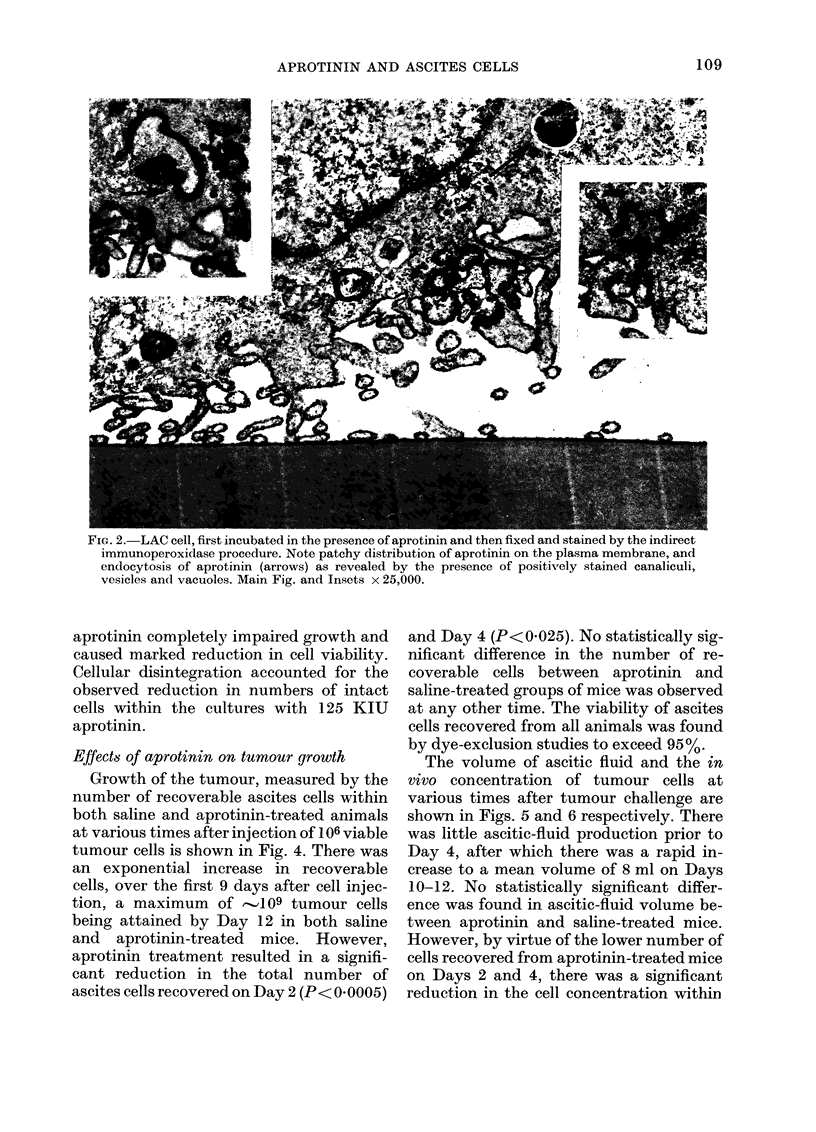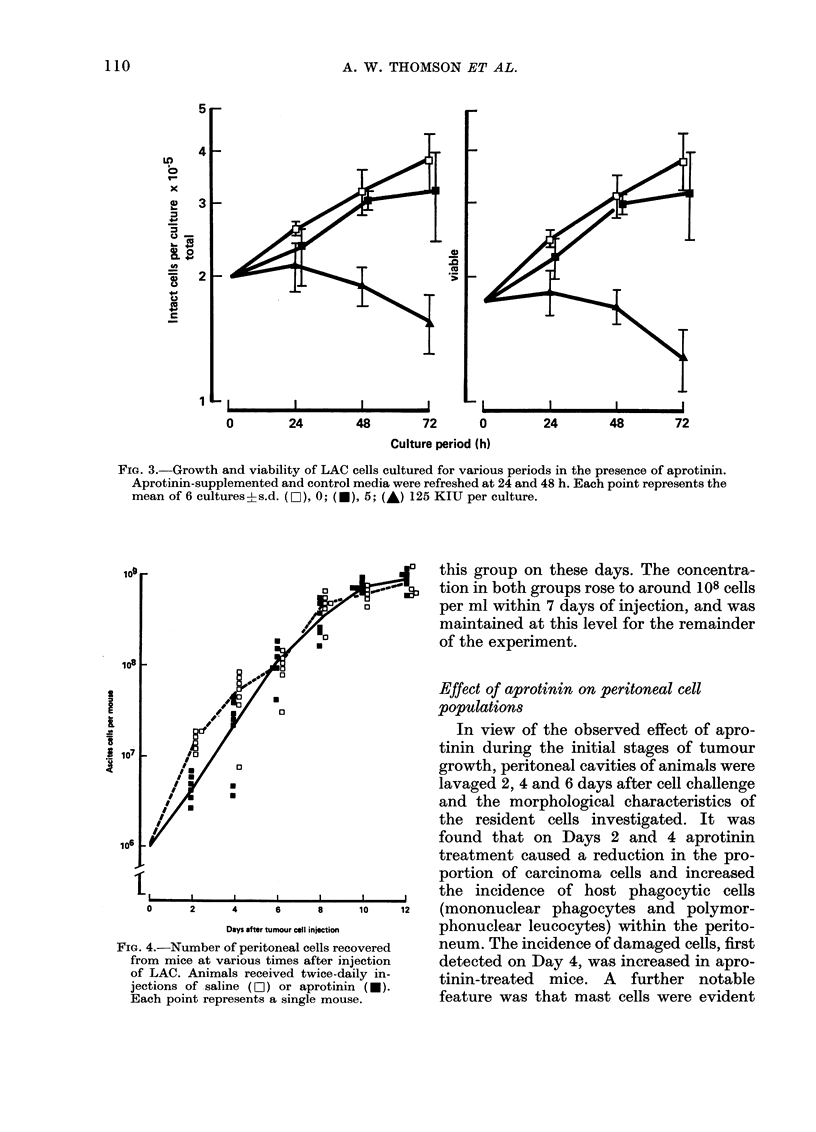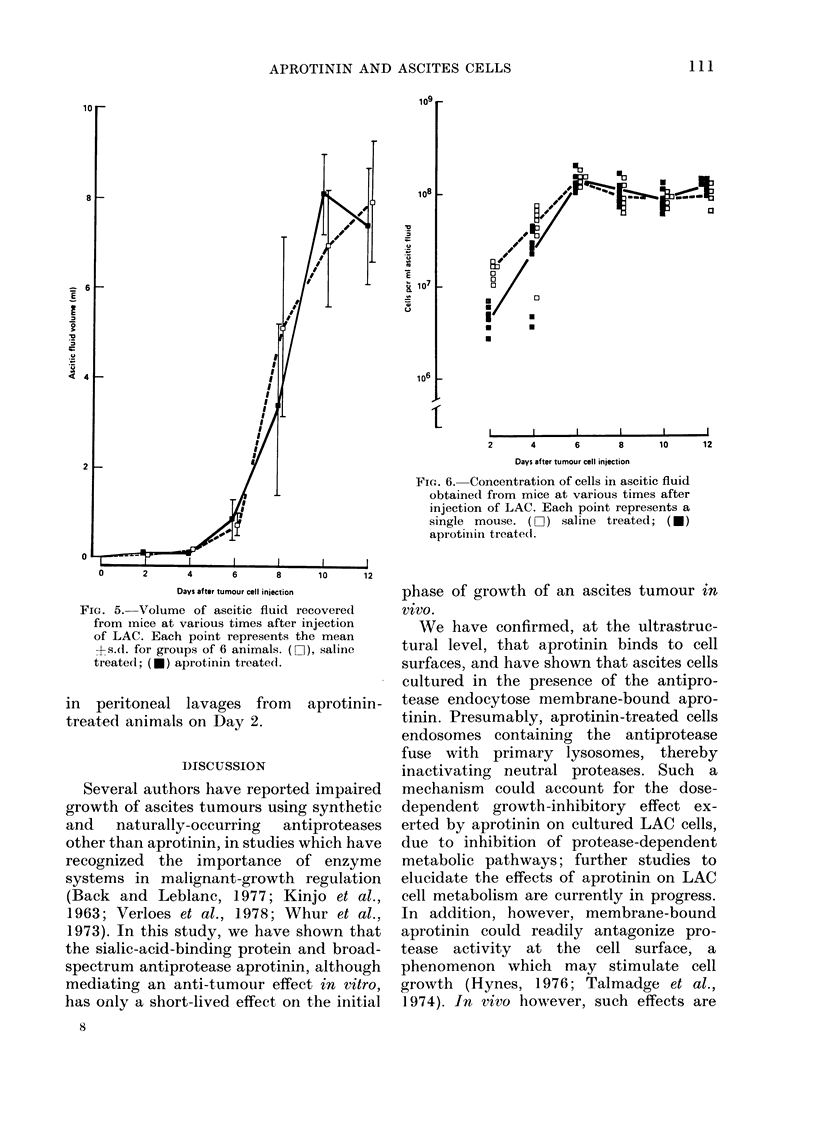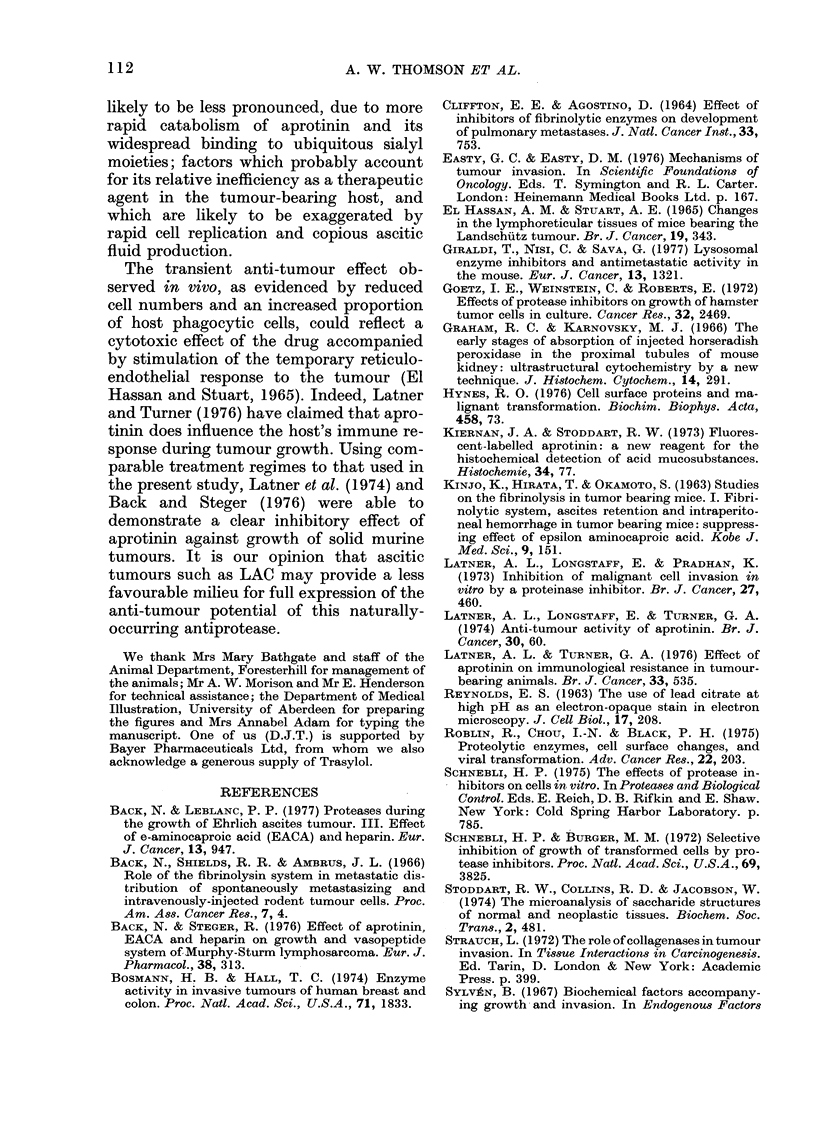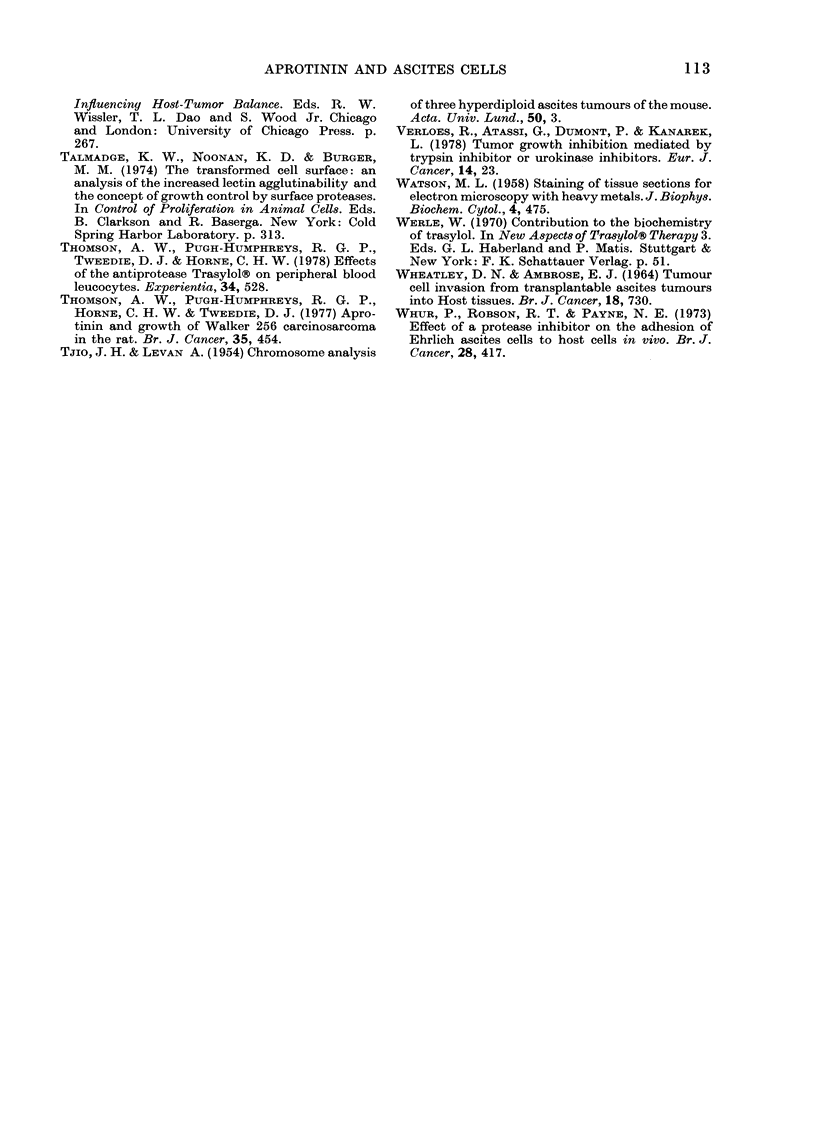# Endocytosis of the antiprotease aprotinin by Landschütz ascites carcinoma cells and its effects in vitro and in vivo.

**DOI:** 10.1038/bjc.1978.169

**Published:** 1978-07

**Authors:** A. W. Thomson, D. J. Tweedie, R. G. Pugh-Humphreys, M. Arthur

## Abstract

**Images:**


*Br. J. Cancer* (1978) **38**, 106

### ENDOCYTOSIS OF THE ANTIPROTEASE APROTININ BY LANDSCHÜTZ ASCITES CARCINOMA CELLS AND ITS EFFECTS *IN VITRO* AND *IN VIVO*

A. W. THOMSON, D. J. TWEEDIE, R. G. P. PUGH-HUMPHREYS* AND M. ARTHUR

*From the Departments of Pathology and Surgery, University Medical Buildings, Foresterhill, Aberdeen and *Department of Zoology, University of Aberdeen*

Received 23 February 1978 Accepted 15 April 1978

**Summary.—Aprotinin was bound and endocytosed by Landschütz ascites carcinoma (LAC) cells *in vitro*. Addition of the antiprotease to cultures of these cells led to a dose-dependent growth-inhibitory and cytotoxic effect. In mice inoculated with LAC cells and treated with aprotinin there was a transient reduction in both the number and concentration of recovered ascites cells during the early phase of tumour growth. This was accompanied by a temporary increase in the proportion of peritoneal phagocytes (mononuclear phagocytes and polymorphonuclear leucocytes) relative to carcinoma cells. However, the number and concentration of ascites cells eventually achieved was comparable in saline and aprotinin-treated animals.**

THE low-molecular-weight polypeptide aprotinin (Trasylol®) which is isolated from bovine lung, binds specifically to sialyl residues, and has been used to demonstrate their presence on the surfaces of a variety of animal cells (Kiernan and Stoddart, 1973) including human lymphocytes (Stoddart *et al.*, 1974). In addition to its carbohydrate-binding properties, aprotinin is a broad-spectrum antiprotease which inhibits the enzymes trypsin, chymotrypsin and plasma kallikrein (Werle, 1970).

Of the wide range of naturally-occurring and synthetic protease inhibitors currently available, some, including aprotinin, impair the growth of malignant cells and their non-malignant homologues *in vitro* (Goetz *et al.*, 1972; Latner *et al.*, 1973; Roblin *et al.*, 1975). Thus, although the original claim of Schnebli and Burger (1972) that the growth-inhibitory effects of protease inhibitors are directed selectively against malignant tumour cells, has not been substantiated (Hynes, 1976; Schnebli, 1975) nevertheless, inhibition of malignant cell growth by protease inhibitors lends to the view (Bosmann and Hall; 1974; Easty and Easty, 1976; Hynes, 1976; Schnebli, 1975; Sylvén, 1967) that tumour cell proteases potentiate the growth of malignant tumours. However, evaluation of aprotinin as an anti-tumour agent *in vivo* by various authors has produced variable results (Back *et al.*, 1966; Back and Steger, 1976; Cliffton and Agostino, 1964; Giraldi *et al.*, 1977; Latner *et al.*, 1974; Thomson *et al.*, 1977).

In this study, we have used an indirect immunoperoxidase technique to examine the binding of aprotinin by Landschütz ascites carcinoma (LAC) cells. Also, in view of reports that a natural protease inhibitor (soybean trypsin inhibitor) impairs growth of ascites tumours (Whur *et al.*, 1973; Verloes *et al.*, 1978) we have attempted to determine whether aprotinin

Correspondence: Dr A. W. Thomson, Department of Pathology, University Medical Buildings, Foresterhill, Aberdeen AB9 2ZD.

exerts similar effects against LAC *in vitro* and *in vivo*.

### MATERIALS AND METHODS

*Mice*.—Closed-colony bred, 5–8-week-old, female LACA mice, ranging in weight from 17 to 24 g were used throughout.

*Aprotinin*.—Aprotinin (Trasylol®) was supplied as a solution (1·5 mg/ml, in 0·9% w/v aqueous NaCl) as marketed for clinical use by Bayer Pharmaceuticals, Haywards Heath, Sussex at an activity of 10,000 kallikrein-inactivating units (KIU) per ml.

*Tumour cells*.—The non-strain-specific Landschütz ascites tumour, a subline of the Ehrlich diploid carcinoma (Tjio and Levan, 1954) was propagated by i.p. passage in female LACA mice. Cells aspirated from the peritoneal cavity on the 7th day of tumour development were washed $\times 3$ in ice-cold Eagle's minimal essential medium (MEM; Wellcome Reagents Limited) and cell viability assessed by trypan-blue exclusion prior to injection.

*Binding of aprotinin*.—Washed ascites cells were cultured within Falcon tissue-culture flasks (Flow Laboratories) in MEM supplemented with 10% foetal bovine serum (Gibco Bio-Cult) for 4 h at 37°C in an atmosphere of 5% $CO_2$ in air. They were then either fixed in 2·5% glutaraldehyde before incubation for 1 h in aprotinin (10,000 KIU/ml) at 20°C, or alternatively, incubated in aprotinin-containing medium (5,000 KIU/ml) and then fixed in glutaraldehyde. In each instance the treated, washed cells were further incubated, first with rabbit anti-aprotinin antibody (1:10; Thomson *et al*., 1978) and then, following several rinses in phosphate buffer, with peroxidase-conjugated goat anti-rabbit serum (Dakopatts A/S). The cytochemical procedure of Graham and Karnovsky (1966) was used to reveal the sites of cell-bound peroxidase-conjugated antibody and the cells then post-fixed for 1 h at 4°C in 1% osmium tetroxide, followed by dehydration in graded ethanols and embedding in TAAB epoxy resin. Ultrathin sections were stained in uranyl acetate (Watson, 1958) and lead citrate (Reynolds, 1963) prior to examination in an AEI EM6B transmission electron microscope.

In vitro *growth and viability studies*.—Two dilutions of aprotinin (5 and 125 KIU) were added to cultures of ascites cells ($2 \times 10^5$ cells in 0·2 ml) in MEM supplemented with 10% heat-inactivated foetal bovine serum within flat-bottomed microcultures plates (3040 Falcon Plastics). Each treatment was replicated 6 times. At 24 and 48 h the culture supernatants were replaced by fresh medium containing equivalent doses of aprotinin to those added at the start of the culture. Cell numbers were estimated by haemocytometry and viability assessed by trypan-blue exclusion at 24, 48 and 72 h.

In vivo *experiments*.—Growth of the tumour was measured in animals injected with $10^6$ viable ascites cells. Each group received either 0·5 ml aprotinin (5,000 KIU) or an equivalent volume of Dulbecco "A" phosphate buffered saline (PBS) i.p. at the same time as tumour injection and twice daily thereafter. At various times after cell challenge 12 mice, 6 from each treatment group, were killed and growth measurements conducted.

*Monitoring of tumour growth*.—Mice were killed by terminal ether anaesthesia. At early stages of tumour development (Days 2 and 4) when there was little measurable ascitic fluid, free ascites cells were removed by lavaging the peritoneal cavity with 10 ml PBS containing heparin (2 IU/ml). At later stages, a midline incision was made in the abdominal wall, the ascitic fluid withdrawn and placed in a pre-weighed test tube. The remaining fluid was removed by swabbing the peritoneal cavity with pre-weighed cotton wool. Since the sp. gr. of ascitic fluid approximates closely to 1 (Wheatley and Ambrose, 1964) the weight of fluid (measured correct to $10^{-3}$ g) corresponded to the volume (ml) of fluid present. Confirmation of ascitic-fluid volume was achieved by weighing the mice (correct to $10^{-2}$ g) before and after removal of the tumour. Total free ascites cells was then given by the product of ascitic fluid volume and the cell count per ml, which, together with cell viability was estimated by haemocytometry.

*Characterization of peritoneal cells*.—Peritoneal cavities of mice injected i.p. with tumour cells, and either aprotinin or saline, were lavaged with heparin-containing medium and the aspirates centrifuged to obtain cell pellets which were fixed in glutaraldehyde followed by osmium tetroxide and then embedded in TAAB epoxy resin. Cytodiagnostic observations on the cells within the pellets together with counts of the different cell types present, were made on 1 $\mu$m sections stained with toluidine blue.

FIG. 1.—Glutaraldehyde-fixed LAC cell stained by the indirect immunoperoxidase procedure to reveal binding of aprotinin to the cell surface. × 8,500. Inset: detail, showing positive staining for aprotinin bound to the outer osmiophilic leaflet of the plasma membrane. × 50,000.

### RESULTS

### *Binding of aprotinin*

Using the indirect immunoperoxidase staining procedure to localise cell-bound aprotinin, an electron-dense precipitate was observed at the surfaces of LAC cells which had been fixed in glutaraldehyde prior to incubation in the protease inhibitor (Fig. 1). The electron-dense precipitate which was some 30–50 nm wide, was adherent to the outer osmiophilic "leaflet" (zone?) of the plasma membrane, revealing that aprotinin had bound to moieties in the glycocalyx region of the tumour-cell surface (Inset, Fig. 1). There was no surface staining visible on those cells which had not been incubated in aprotinin prior to the indirect immunoperoxidase staining.

Ultrastructural studies on LAC cells which had been incubated in medium containing aprotinin prior to fixation and indirect immunoperoxidase staining (Fig. 2) revealed not only binding of aprotinin to the surface membranes of the tumour cells, but also the presence of numerous positively stained intracellular vermiform canaliculi, vesicles and vacuoles (Insets, Fig. 2) which indicated that the tumour cells had endocytosed aprotinin, a factor which probably accounts for the relatively patchy distribution of the surface staining for aprotinin observed on these cells compared with that observed on the surfaces of the glutaraldehyde-prefixed ascites tumour cells.

### *Effect of aprotinin on cell growth and viability* in vitro

Fig. 3 shows the growth and viability of ascites cells cultured in the presence of aprotinin. A dose-dependent cytotoxic and growth-inhibitory effect was observed after 24, 48 and 72 h. Addition of 125 KIU

FIG. 2.—LAC cell, first incubated in the presence of aprotinin and then fixed and stained by the indirect immunoperoxidase procedure. Note patchy distribution of aprotinin on the plasma membrane, and endocytosis of aprotinin (arrows) as revealed by the presence of positively stained canaliculi, vesicles and vacuoles. Main Fig. and Insets ×25,000.

aprotinin completely impaired growth and caused marked reduction in cell viability. Cellular disintegration accounted for the observed reduction in numbers of intact cells within the cultures with 125 KIU aprotinin.

*Effects of aprotinin on tumour growth*

Growth of the tumour, measured by the number of recoverable ascites cells within both saline and aprotinin-treated animals at various times after injection of $10^6$ viable tumour cells is shown in Fig. 4. There was an exponential increase in recoverable cells, over the first 9 days after cell injection, a maximum of $\sim 10^9$ tumour cells being attained by Day 12 in both saline and aprotinin-treated mice. However, aprotinin treatment resulted in a significant reduction in the total number of ascites cells recovered on Day 2 ($P < 0{\cdot}0005$) and Day 4 ($P < 0{\cdot}025$). No statistically significant difference in the number of recoverable cells between aprotinin and saline-treated groups of mice was observed at any other time. The viability of ascites cells recovered from all animals was found by dye-exclusion studies to exceed 95%.

The volume of ascitic fluid and the *in vivo* concentration of tumour cells at various times after tumour challenge are shown in Figs. 5 and 6 respectively. There was little ascitic-fluid production prior to Day 4, after which there was a rapid increase to a mean volume of 8 ml on Days 10–12. No statistically significant difference was found in ascitic-fluid volume between aprotinin and saline-treated mice. However, by virtue of the lower number of cells recovered from aprotinin-treated mice on Days 2 and 4, there was a significant reduction in the cell concentration within

FIG. 3.—Growth and viability of LAC cells cultured for various periods in the presence of aprotinin. Aprotinin-supplemented and control media were refreshed at 24 and 48 h. Each point represents the mean of 6 cultures ± s.d. (□), 0; (■), 5; (▲) 125 KIU per culture.

FIG. 4.—Number of peritoneal cells recovered from mice at various times after injection of LAC. Animals received twice-daily injections of saline (□) or aprotinin (■). Each point represents a single mouse.

this group on these days. The concentration in both groups rose to around $10^8$ cells per ml within 7 days of injection, and was maintained at this level for the remainder of the experiment.

*Effect of aprotinin on peritoneal cell populations*

In view of the observed effect of aprotinin during the initial stages of tumour growth, peritoneal cavities of animals were lavaged 2, 4 and 6 days after cell challenge and the morphological characteristics of the resident cells investigated. It was found that on Days 2 and 4 aprotinin treatment caused a reduction in the proportion of carcinoma cells and increased the incidence of host phagocytic cells (mononuclear phagocytes and polymorphonuclear leucocytes) within the peritoneum. The incidence of damaged cells, first detected on Day 4, was increased in aprotinin-treated mice. A further notable feature was that mast cells were evident

FIG. 5.—Volume of ascitic fluid recovered from mice at various times after injection of LAC. Each point represents the mean ±s.d. for groups of 6 animals. (□), saline treated; (■) aprotinin treated.

FIG. 6.—Concentration of cells in ascitic fluid obtained from mice at various times after injection of LAC. Each point represents a single mouse. (□) saline treated; (■) aprotinin treated.

in peritoneal lavages from aprotinin-treated animals on Day 2.

### DISCUSSION

Several authors have reported impaired growth of ascites tumours using synthetic and naturally-occurring antiproteases other than aprotinin, in studies which have recognized the importance of enzyme systems in malignant-growth regulation (Back and Leblanc, 1977; Kinjo *et al.*, 1963; Verloes *et al.*, 1978; Whur *et al.*, 1973). In this study, we have shown that the sialic-acid-binding protein and broad-spectrum antiprotease aprotinin, although mediating an anti-tumour effect *in vitro*, has only a short-lived effect on the initial phase of growth of an ascites tumour *in vivo*.

We have confirmed, at the ultrastructural level, that aprotinin binds to cell surfaces, and have shown that ascites cells cultured in the presence of the antiprotease endocytose membrane-bound aprotinin. Presumably, aprotinin-treated cells endosomes containing the antiprotease fuse with primary lysosomes, thereby inactivating neutral proteases. Such a mechanism could account for the dose-dependent growth-inhibitory effect exerted by aprotinin on cultured LAC cells, due to inhibition of protease-dependent metabolic pathways; further studies to elucidate the effects of aprotinin on LAC cell metabolism are currently in progress. In addition, however, membrane-bound aprotinin could readily antagonize protease activity at the cell surface, a phenomenon which may stimulate cell growth (Hynes, 1976; Talmadge *et al.*, 1974). *In vivo* however, such effects are likely to be less pronounced, due to more rapid catabolism of aprotinin and its widespread binding to ubiquitous sialyl moieties; factors which probably account for its relative inefficiency as a therapeutic agent in the tumour-bearing host, and which are likely to be exaggerated by rapid cell replication and copious ascitic fluid production.

The transient anti-tumour effect observed *in vivo*, as evidenced by reduced cell numbers and an increased proportion of host phagocytic cells, could reflect a cytotoxic effect of the drug accompanied by stimulation of the temporary reticuloendothelial response to the tumour (El Hassan and Stuart, 1965). Indeed, Latner and Turner (1976) have claimed that aprotinin does influence the host's immune response during tumour growth. Using comparable treatment regimes to that used in the present study, Latner *et al.* (1974) and Back and Steger (1976) were able to demonstrate a clear inhibitory effect of aprotinin against growth of solid murine tumours. It is our opinion that ascitic tumours such as LAC may provide a less favourable milieu for full expression of the anti-tumour potential of this naturally-occurring antiprotease.

We thank Mrs Mary Bathgate and staff of the Animal Department, Foresterhill for management of the animals; Mr A. W. Morison and Mr E. Henderson for technical assistance; the Department of Medical Illustration, University of Aberdeen for preparing the figures and Mrs Annabel Adam for typing the manuscript. One of us (D.J.T.) is supported by Bayer Pharmaceuticals Ltd, from whom we also acknowledge a generous supply of Trasylol.

### REFERENCES

BACK, N. & LEBLANC, P. P. (1977) Proteases during the growth of Ehrlich ascites tumour. III. Effect of e-aminocaproic acid (EACA) and heparin. *Eur. J. Cancer*, **13**, 947.

BACK, N., SHIELDS, R. R. & AMBRUS, J. L. (1966) Role of the fibrinolysin system in metastatic distribution of spontaneously metastasizing and intravenously-injected rodent tumour cells. *Proc. Am. Ass. Cancer Res.*, **7**, 4.

BACK, N. & STEGER, R. (1976) Effect of aprotinin, EACA and heparin on growth and vasopeptide system of Murphy-Sturm lymphosarcoma. *Eur. J. Pharmacol.*, **38**, 313.

BOSMANN, H. B. & HALL, T. C. (1974) Enzyme activity in invasive tumours of human breast and colon. *Proc. Natl. Acad. Sci., U.S.A.*, **71**, 1833.

CLIFFTON, E. E. & AGOSTINO, D. (1964) Effect of inhibitors of fibrinolytic enzymes on development of pulmonary metastases. *J. Natl. Cancer Inst.*, **33**, 753.

EASTY, G. C. & EASTY, D. M. (1976) Mechanisms of tumour invasion. In *Scientific Foundations of Oncology*. Eds. T. Symington and R. L. Carter. London: Heinemann Medical Books Ltd. p. 167.

EL HASSAN, A. M. & STUART, A. E. (1965) Changes in the lymphoreticular tissues of mice bearing the Landschütz tumour. *Br. J. Cancer*, **19**, 343.

GIRALDI, T., NISI, C. & SAVA, G. (1977) Lysosomal enzyme inhibitors and antimetastatic activity in the mouse. *Eur. J. Cancer*, **13**, 1321.

GOETZ, I. E., WEINSTEIN, C. & ROBERTS, E. (1972) Effects of protease inhibitors on growth of hamster tumor cells in culture. *Cancer Res.*, **32**, 2469.

GRAHAM, R. C. & KARNOVSKY, M. J. (1966) The early stages of absorption of injected horseradish peroxidase in the proximal tubules of mouse kidney: ultrastructural cytochemistry by a new technique. *J. Histochem. Cytochem.*, **14**, 291.

HYNES, R. O. (1976) Cell surface proteins and malignant transformation. *Biochim. Biophys. Acta*, **458**, 73.

KIERNAN, J. A. & STODDART, R. W. (1973) Fluorescent-labelled aprotinin: a new reagent for the histochemical detection of acid mucosubstances. *Histochemie*, **34**, 77.

KINJO, K., HIRATA, T. & OKAMOTO, S. (1963) Studies on the fibrinolysis in tumor bearing mice. I. Fibrinolytic system, ascites retention and intraperitoneal hemorrhage in tumor bearing mice: suppressing effect of epsilon aminocaproic acid. *Kobe J. Med. Sci.*, **9**, 151.

LATNER, A. L., LONGSTAFF, E. & PRADHAN, K. (1973) Inhibition of malignant cell invasion *in vitro* by a proteinase inhibitor. *Br. J. Cancer*, **27**, 460.

LATNER, A. L., LONGSTAFF, E. & TURNER, G. A. (1974) Anti-tumour activity of aprotinin. *Br. J. Cancer*, **30**, 60.

LATNER, A. L. & TURNER, G. A. (1976) Effect of aprotinin on immunological resistance in tumour-bearing animals. *Br. J. Cancer*, **33**, 535.

REYNOLDS, E. S. (1963) The use of lead citrate at high pH as an electron-opaque stain in electron microscopy. *J. Cell Biol.*, **17**, 208.

ROBLIN, R., CHOU, I.-N. & BLACK, P. H. (1975) Proteolytic enzymes, cell surface changes, and viral transformation. *Adv. Cancer Res.*, **22**, 203.

SCHNEBLI, H. P. (1975) The effects of protease inhibitors on cells *in vitro*. In *Proteases and Biological Control*. Eds. E. Reich, D. B. Rifkin and E. Shaw. New York: Cold Spring Harbor Laboratory. p. 785.

SCHNEBLI, H. P. & BURGER, M. M. (1972) Selective inhibition of growth of transformed cells by protease inhibitors. *Proc. Natl. Acad. Sci., U.S.A.*, **69**, 3825.

STODDART, R. W., COLLINS, R. D. & JACOBSON, W. (1974) The microanalysis of saccharide structures of normal and neoplastic tissues. *Biochem. Soc. Trans.*, **2**, 481.

STRAUCH, L. (1972) The role of collagenases in tumour invasion. In *Tissue Interactions in Carcinogenesis*. Ed. Tarin, D. London & New York: Academic Press. p. 399.

SYLVÉN, B. (1967) Biochemical factors accompanying growth and invasion. In *Endogenous Factors*

*Influencing Host-Tumor Balance*. Eds. R. W. Wissler, T. L. Dao and S. Wood Jr. Chicago and London: University of Chicago Press. p. 267.

TALMADGE, K. W., NOONAN, K. D. & BURGER, M. M. (1974) The transformed cell surface: an analysis of the increased lectin agglutinability and the concept of growth control by surface proteases. In *Control of Proliferation in Animal Cells*. Eds. B. Clarkson and R. Baserga. New York: Cold Spring Harbor Laboratory. p. 313.

THOMSON, A. W., PUGH-HUMPHREYS, R. G. P., TWEEDIE, D. J. & HORNE, C. H. W. (1978) Effects of the antiprotease Trasylol® on peripheral blood leucocytes. *Experientia*, **34**, 528.

THOMSON, A. W., PUGH-HUMPHREYS, R. G. P., HORNE, C. H. W. & TWEEDIE, D. J. (1977) Aprotinin and growth of Walker 256 carcinosarcoma in the rat. *Br. J. Cancer*, **35**, 454.

TJIO, J. H. & LEVAN A. (1954) Chromosome analysis of three hyperdiploid ascites tumours of the mouse. *Acta. Univ. Lund.*, **50**, 3.

VERLOES, R., ATASSI, G., DUMONT, P. & KANAREK, L. (1978) Tumor growth inhibition mediated by trypsin inhibitor or urokinase inhibitors. *Eur. J. Cancer*, **14**, 23.

WATSON, M. L. (1958) Staining of tissue sections for electron microscopy with heavy metals. *J. Biophys. Biochem. Cytol.*, **4**, 475.

WERLE, W. (1970) Contribution to the biochemistry of trasylol. In *New Aspects of Trasylol® Therapy* 3. Eds. G. L. Haberland and P. Matis. Stuttgart & New York: F. K. Schattauer Verlag. p. 51.

WHEATLEY, D. N. & AMBROSE, E. J. (1964) Tumour cell invasion from transplantable ascites tumours into Host tissues. *Br. J. Cancer*, **18**, 730.

WHUR, P., ROBSON, R. T. & PAYNE, N. E. (1973) Effect of a protease inhibitor on the adhesion of Ehrlich ascites cells to host cells *in vivo*. *Br. J. Cancer*, **28**, 417.